# Hepatic mesenchymal hamartoma showing hepatobiliary-phase signal increase on gadoxetate disodium–enhanced magnetic resonance imaging: A case report

**DOI:** 10.1016/j.radcr.2026.07.126

**Published:** 2026-09-01

**Authors:** Tsubasa Sugie, Nanae Tsuchiya, Shota Kinjo, Masaaki Kuda, Kennosuke Karube, Kazunari Arakaki, Satoru Hamada, Tokiko Oshiro, Shinobu Kiyuna, Mitsuhisa Takatsuki, Akihiro Nishie

**Affiliations:** a Department of Radiology, Graduate School of Medicine, University of the Ryukyus, Okinawa, Japan; b Department of Digestive and General Surgery, Graduate School of Medicine, University of the Ryukyus, Okinawa, Japan; c Department of Pathology and Laboratory Medicine, Nagoya University Graduate School of Medicine, Nagoya, Japan; d Department of Pathology, Ryukyu University Hospital, Okinawa, Japan; e Department of Child Health and Welfare (Pediatrics), Graduate School of Medicine, University of the Ryukyus, Okinawa, Japan

**Keywords:** Hepatic mesenchymal hamartoma, Gadoxetate disodium, Hepatobiliary phase, Pediatric liver tumor, Organic anion transporting polypeptide 1B3

## Abstract

We report a case of a 9-month-old girl with mesenchymal hamartoma that was evaluated using gadoxetate disodium–enhanced magnetic resonance imaging. Imaging revealed a large hepatic mass with mixed cystic and solid components, with a slight signal increase in the solid component during the hepatobiliary phase. Histopathology confirmed mesenchymal hamartoma, with normal hepatic parenchyma reticularly interspersed within the mass. Immunohistochemical staining for organic anion transporting polypeptide 1B3 showed cytoplasmic immunoreactivity without membranous staining in hepatocyte-like cells. Mesenchymal hamartoma is a rare benign liver tumor in children, accounting for approximately 8% of all pediatric hepatic tumors. This case suggests that mesenchymal hamartoma may show a slight hepatobiliary-phase signal increase, potentially reflecting delayed contrast retention within the interstitium of the solid component.

## Introduction

Mesenchymal hamartoma is a rare benign liver tumor that accounts for approximately 8% of all pediatric liver tumors and is the second most common benign hepatic tumor in children, following infantile hemangioma [1]. Though it is generally considered a benign tumor, in rare cases it may progress to undifferentiated embryonal sarcoma. Although some cases regress spontaneously with time, surgical resection is often performed as treatment, so preoperative imaging such as ultrasound, CT, and MRI is important. However, although unenhanced MRI has been used in some cases for preoperative diagnosis [[2], [3], [4]], few reports have described the use of gadoxetate disodium–enhanced MRI in hepatic mesenchymal hamartoma. We report a case of mesenchymal hamartoma showing a slight hepatobiliary-phase signal increase, with radiologic–pathologic correlation.

## Case presentation

A 9-month-old girl with no relevant personal or family medical history presented to her primary pediatrician with increasing abdominal distention. Physical examination revealed a large abdominal mass. The serum α-fetoprotein level was elevated at 6410 ng/mL (reference value, ≤9 ng/mL). An abdominal CT revealed a large hepatic mass.

**Fig. 1:**
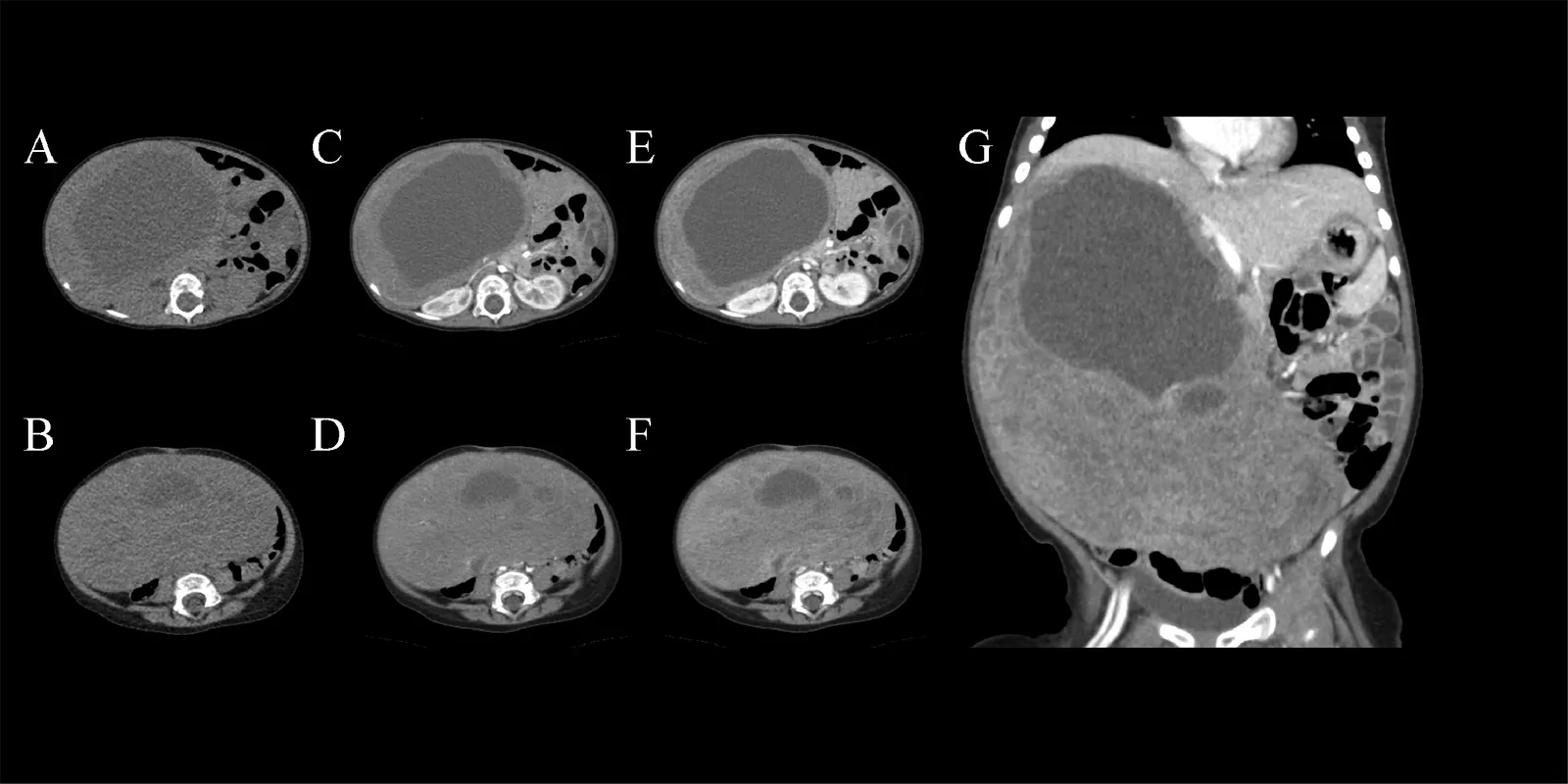
Dynamic contrast-enhanced CT. (A and B): Unenhanced phase, (C and D): Arterial phase, (E and F): Portal phase, (G): Portal phase. Contrast agent was injected at 0.7 mL/s using an injector (for 30 seconds). The arterial phase was captured immediately after injection completion, and the portal phase was captured 20 seconds after contrast agent injection. A large cystic mass extending from the right hepatic lobe into the pelvis was observed. The cystic component measured up to 19 cm in diameter and showed no enhancement. Faint contrast enhancement was noted in the septa and solid portions. No hemorrhage or calcification was observed.

CT findings (Fig. 1): CT revealed a predominantly multicystic mass measuring 19 cm in maximum diameter in the right hepatic lobe, with a solid component in its caudal portion. The septa and solid component showed faint enhancement during the arterial and portal venous phases. No hemorrhage or calcification was observed.

MRI acquisition: MRI was performed using a 1.5-T system (MAGNETOM Avanto, Siemens Healthineers, Erlangen, Germany) with a Body 18 receiver coil. The imaging protocol included axial T2-weighted BLADE imaging (repetition time/echo time, 6480/108 ms), T1-weighted dual-echo in-phase and opposed-phase imaging (170/4.83/2.33 ms; flip angle, 75°), diffusion-weighted imaging (b values, 0 and 800 s/mm²) with apparent diffusion coefficient mapping, and dynamic contrast-enhanced T1-weighted imaging (Fast Low Angle Shot (FLASH); 279/2.38 ms; flip angle, 90°). The slice thickness was 6 mm. Gadoxetate disodium was administered manually through a peripheral intravenous catheter at a dose of 0.1 mL/kg (total volume, 0.87 mL), followed by 10 mL of saline. Dynamic images were acquired 30, 70, and 180 seconds after contrast administration. Hepatobiliary-phase images were obtained 20 min after intravenous administration of gadoxetate disodium, consistent with established pediatric liver MRI protocols, to allow sufficient hepatocellular uptake of the contrast agent [15].

MRI was performed under intravenous sedation supervised by a pediatrician. Sedation was induced with thiopental (5 mg/kg). When the patient awoke approximately 40 min after the start of the examination, an additional dose of midazolam (0.15 mg/kg) was administered to complete the examination.

**Fig. 2:**
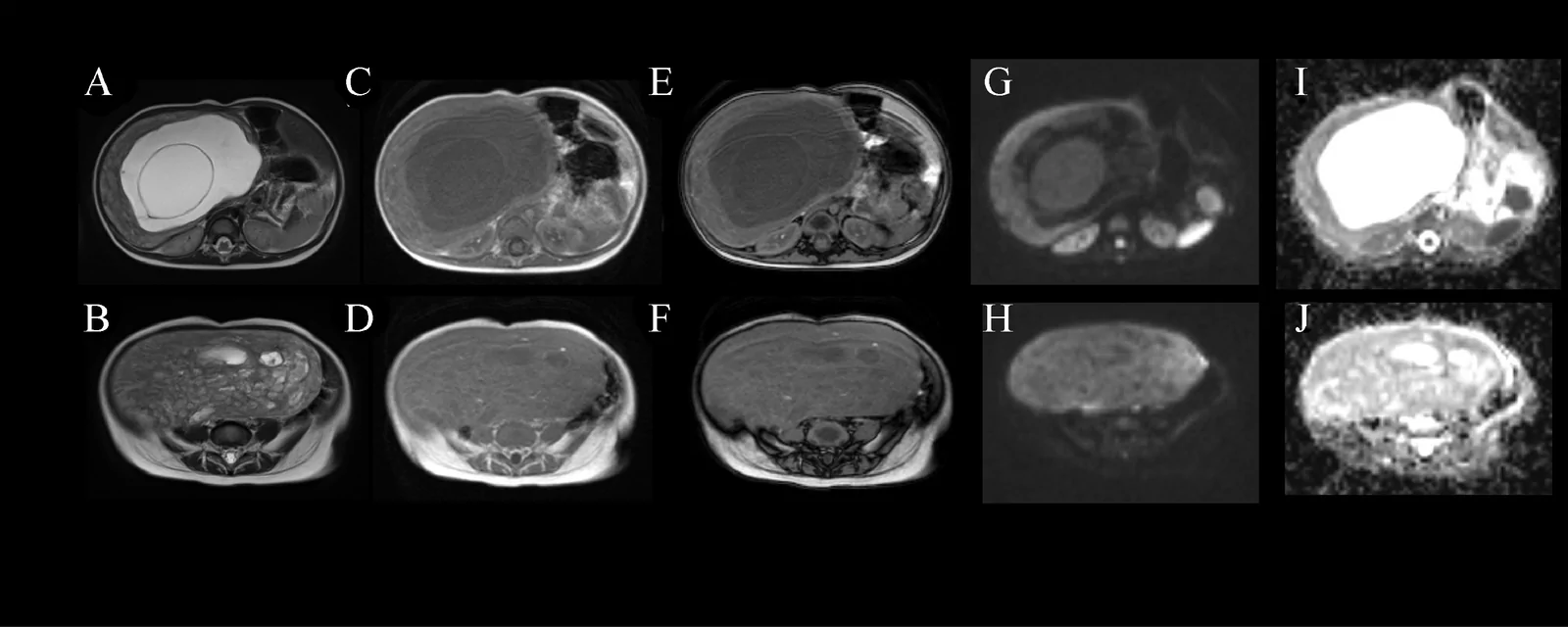
MRI. (A and B) Axial T2-weighted images acquired using the BLADE technique, a motion-resistant radial k-space sampling method; (C and D) axial T1-weighted in-phase images; (E and F) axial T1-weighted opposed-phase images; (G and H) diffusion-weighted images obtained at a b value of 800 s/mm²; and (I and J) apparent diffusion coefficient maps. The MR images were obtained at different axial levels to best demonstrate the cystic and solid components of the tumor. The cystic component shows low signal on T1-weighted imaging (T1WI) and high signal on T2-weighted imaging (T2WI). A solid component demonstrates a heterogeneous signal: low intensity on T1WI and mixed low-to-high signal on T2WI. No restricted diffusion is evident on the diffusion-weighted images or apparent diffusion coefficient maps.

**Fig. 3:**
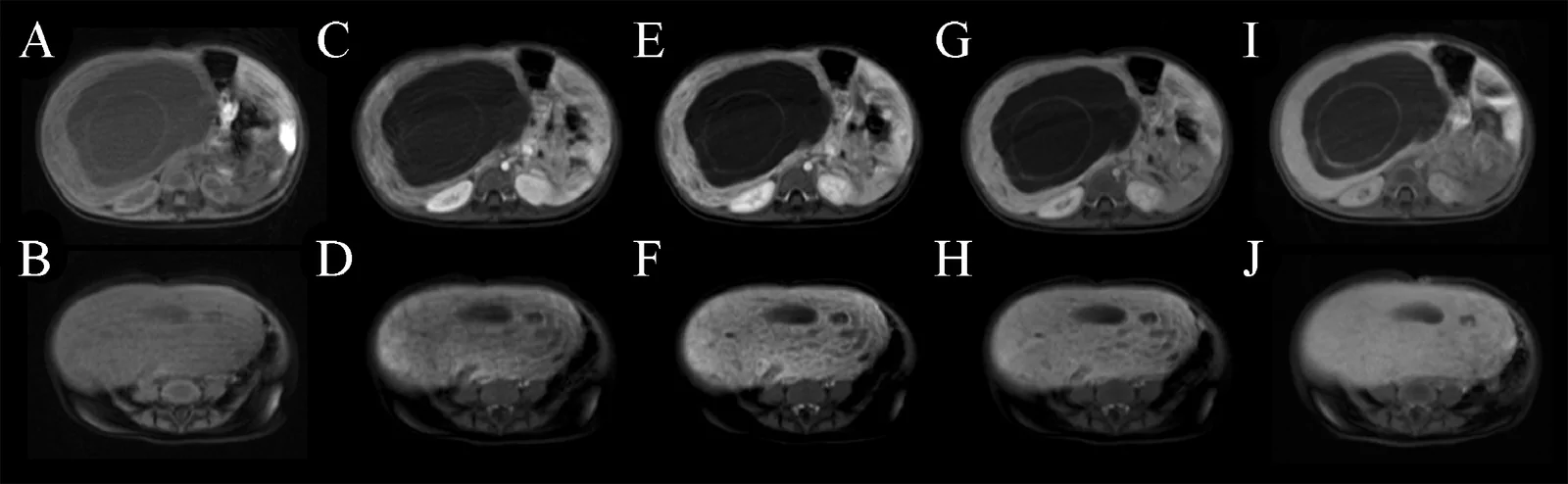
Dynamic gadoxetate disodium–enhanced magnetic resonance imaging. (A and B) Precontrast images; (C and D) 30 seconds after injection; (E and F) 70 seconds after injection; (G and H) 180 seconds after injection; and (I and J) 20 min after injection. Mild contrast enhancement was seen in the septa. The caudal region demonstrated enhancement with slightly lower signal intensity than the normal hepatic parenchyma.

**Fig. 4:**
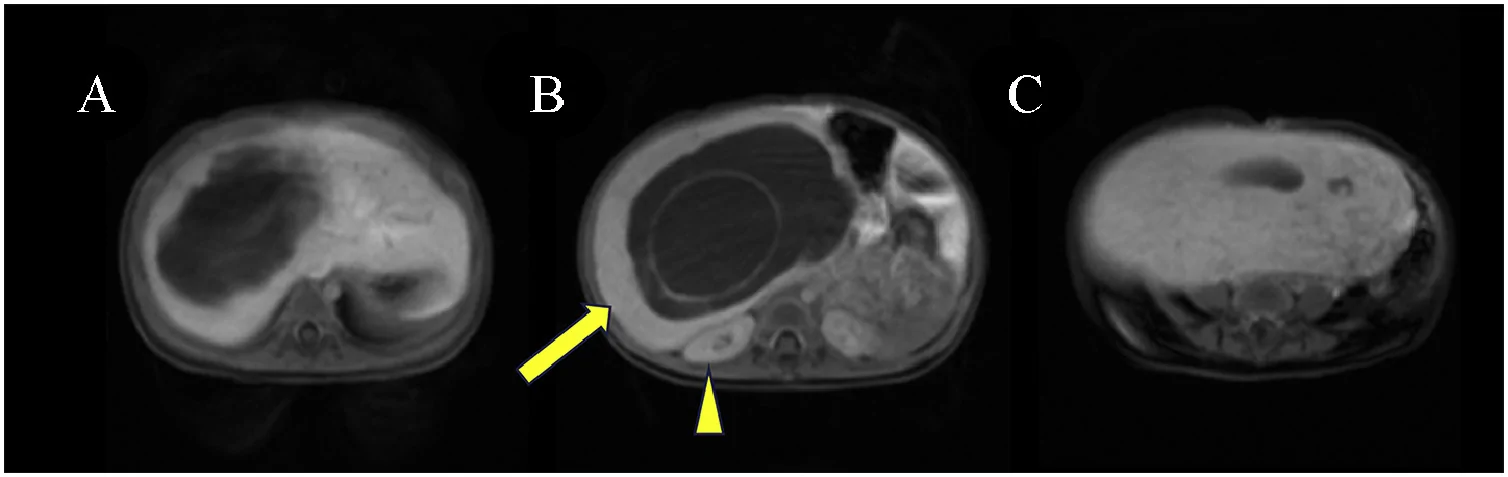
Hepatobiliary-phase gadoxetate disodium–enhanced magnetic resonance imaging. (A-C): Hepatobiliary phase images arrow: solid component; arrowhead: renal cortex. In the hepatobiliary phase, the enhanced areas exhibited lower signal intensity than the normal liver. The solid component shows low signal intensity compared to the renal cortex in precontrast images and exhibits isointense signal in the hepatobiliary phase.

MRI findings (Fig. 2, Fig. 3, Fig. 4): A large mass with mixed cystic and solid components was observed in the right hepatic lobe. The cystic component demonstrated low signal intensity on T1-weighted images and markedly high signal intensity on T2-weighted images. The solid component showed low signal intensity on T1-weighted images and heterogeneous signal intensity ranging from low to high signal on T2-weighted images. Dynamic gadoxetate disodium–enhanced images showed enhancement of the internal septum of the cyst and solid component. During the hepatobiliary phase, the solid component was slightly hypointense relative to the normal liver parenchyma and isointense to the renal cortex. Because the solid component was hypointense relative to the renal cortex on the precontrast images, this change indicated a slight hepatobiliary-phase signal increase. There was no obvious diffusion restriction or fatty component in the mass (Fig. 3).

Preoperative course: A biopsy was performed under the right costal arch. Because hepatoblastoma could not be excluded based on the markedly elevated serum AFP level, chemotherapy was initiated while the biopsy results were pending. The patient received 2 courses of CITA chemotherapy (cisplatin, pirarubicin). After the biopsy established the diagnosis of mesenchymal hamartoma, chemotherapy was discontinued. Follow-up imaging performed 1 month after chemotherapy showed a decrease in the maximum tumor diameter from 19 cm to 16 cm, with reduced compression of the hepatic veins, portal vein, and adjacent abdominal organs. No substantial change was observed in the internal imaging characteristics of the tumor. The pathological diagnosis based on the biopsy specimen was mesenchymal hamartoma of the liver. Right hepatectomy was performed.

Operative findings: The liver was a well-defined surface and sharp, soft margins. The tumor was a large mass extending from the right lobe of the liver to the midline and the left side of the pelvis, with a slightly irregular surface, clear borders, and elastic softness without adhesions. The right lobectomy of the liver was performed, and the tumor was successfully removed.

**Fig. 5:**
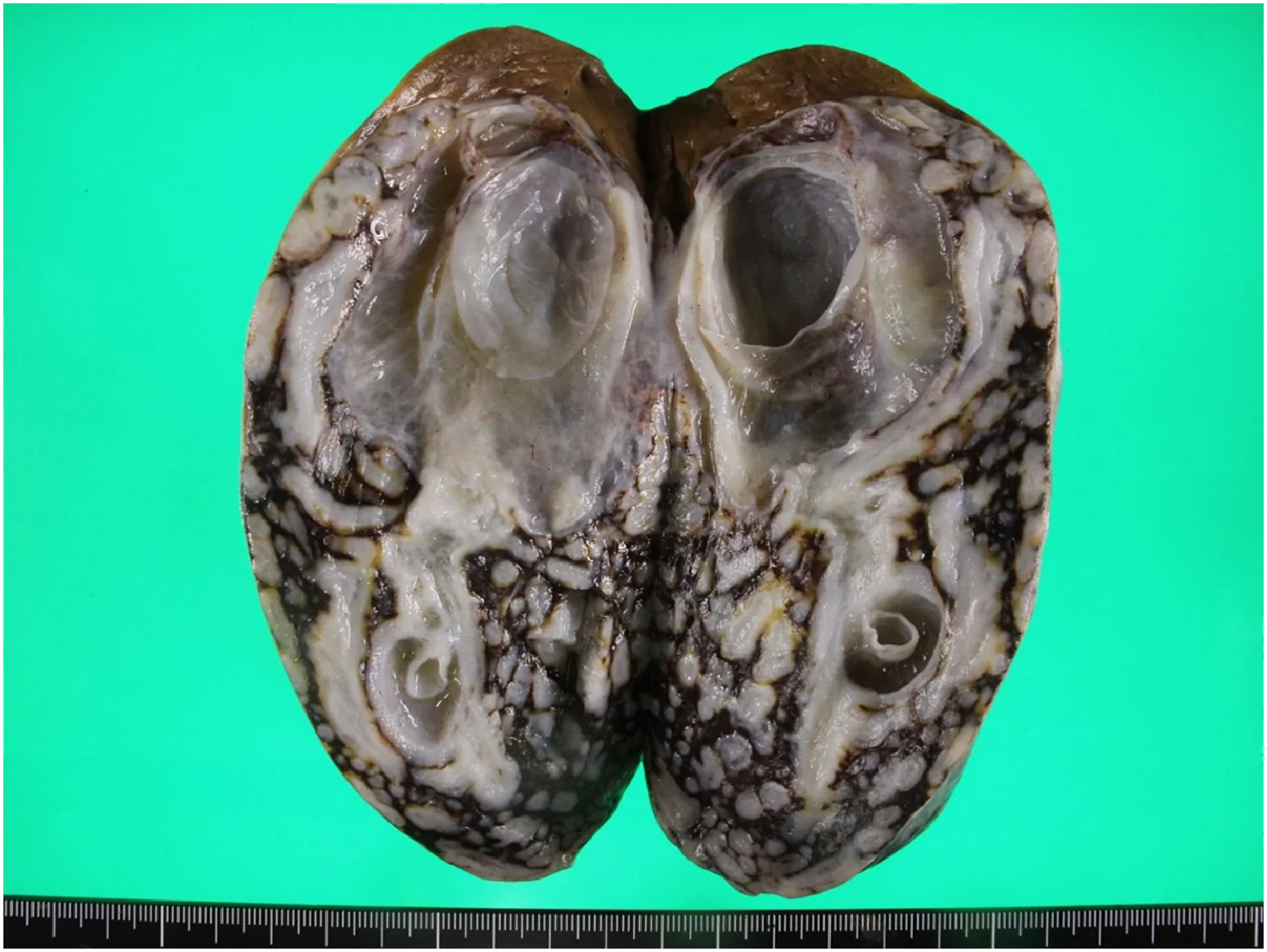
The lesion included denuded bile duct-lined cystic portions and solid, whitish fibrous areas.

**Fig. 6:**
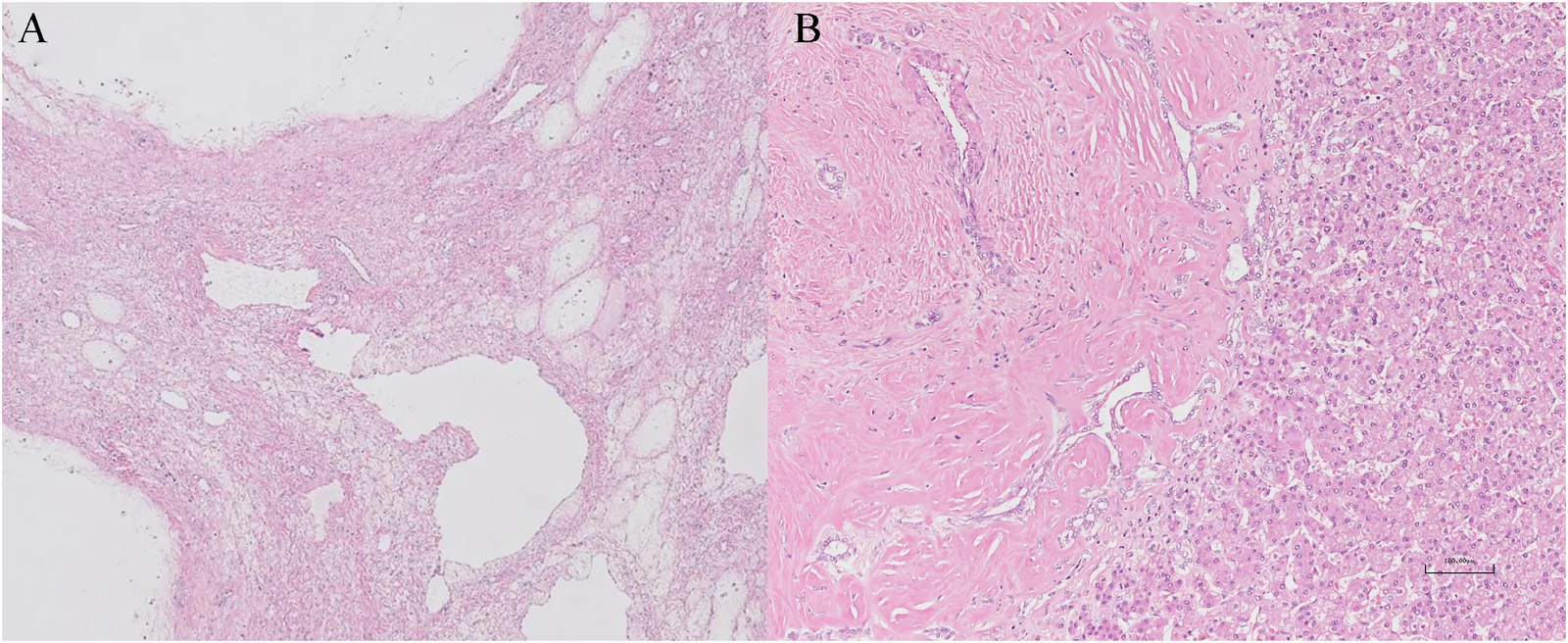
HE staining. (A): Cystic component, (B): Solid component. Degenerated bile ducts with desquamated epithelium, surrounding myxedema-like stroma, and dilated lymphatic vessels. Fibrosis is seen on the left side of the image and normal liver tissue on the right; no dysplastic cells were observed.

**Fig. 7:**
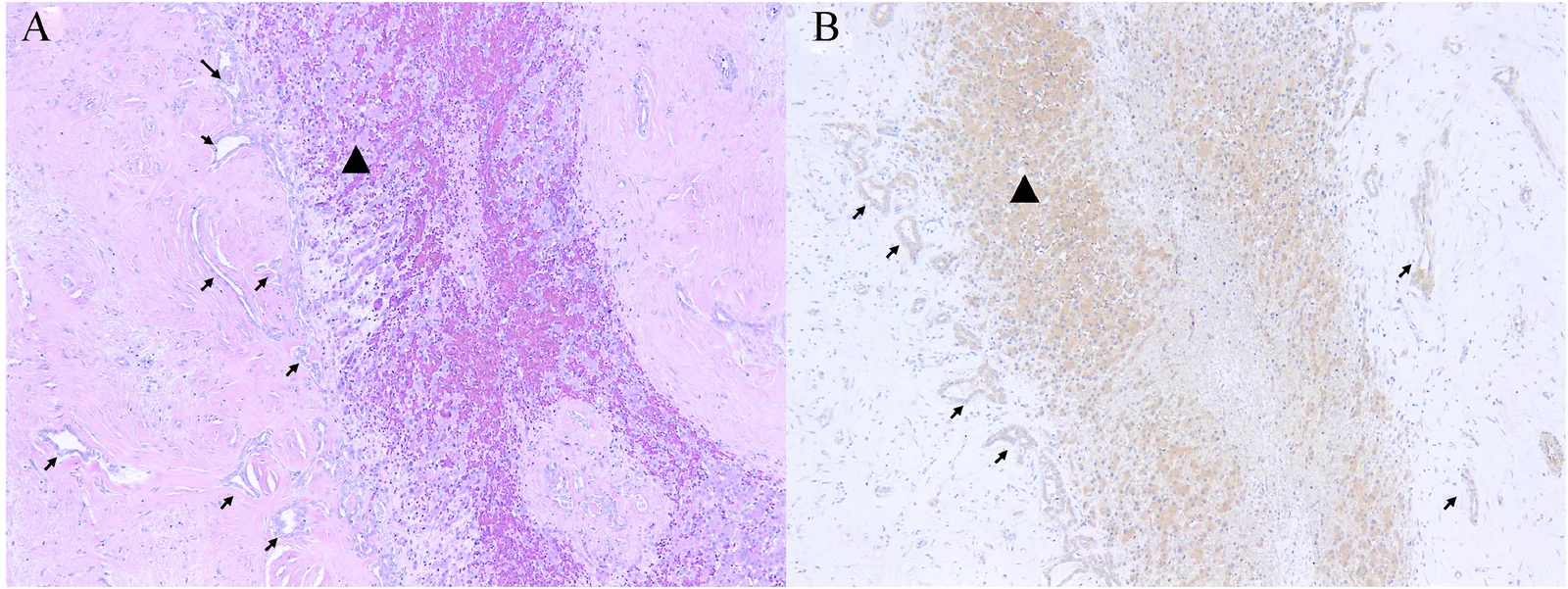
OATP immunohistochemistry. (A and B): ▲: Differentiation toward hepatocytes; →: bile duct-like structures. Bile ducts were pronounced and stained positive; interstitial components were negative. Hepatocyte components were positive in the cytoplasm but negative on the cell membrane.

Pathological findings (Fig. 5, Fig. 6, Fig. 7): The right lobe contained a tumor featuring small and large cystic components with pale yellow transparent serous to mucous fluid and exhibiting white tone, with diffuse growth of islands arising from the cysts. Histologically, there was a myxomatous to fibrotic stroma around the small bile ducts in the tumor area, and bile duct hyperplasia was seen at the margins. Normal liver parenchyma was reticularly interspersed within the mass. No extramedullary hematopoiesis was observed. Additional AFP staining was negative for hepatocyte, bile duct, and interstitial components; OATP-1B3 staining was positive for bile duct components and negative for interstitial components; hepatocyte components were positive in the cytoplasm and negative in the cell membrane (Fig. 6).

Postoperative course: The patient was discharged without any significant complications. AFP levels decreased after tumor resection, and there has been no tumor recurrence 4 years after the surgery.

## Discussion

Mesenchymal hamartoma is a rare hepatic tumor that accounts for approximately 8% of all pediatric liver tumors and is the second most common benign primary hepatic tumor in children after infantile hemangioma [1]. The age of onset ranges from neonatal period to 10 years, but it usually occurs within the first 2 years of life, with peak incidence between 10 and 20 months [5]. Approximately 80% of cases are diagnosed before 2 years of age and 95% before 5 years of age [6], and the tumors are more common in boys. It may be found as an asymptomatic abdominal mass after birth, but larger lesions may present with symptoms such as abdominal distention, dyspnea associated with elevation of the diaphragm, and anorexia. They range in size from 3 to 21 cm and are often very large, 10 cm or more (average, 16 cm), with a predilection for the right lobe [7]. It is considered a developmental malformation rather than a true neoplasm and is morphologically composed of fibrous hard parenchyma and small and large cysts. The ratio of the solid component to the cystic component varies from case to case, but it is estimated that 70% of all cases show a cystic component. The cysts contain a mixture of bile ducts, hepatocytes, vascular structures, and immature mesenchymal tissue. When only a solid component is present, distinguishing mesenchymal hamartoma from malignant liver tumors becomes important. Differential diagnosis in patients of a similar age include undifferentiated sarcoma, hepatoblastoma, hemangioendothelioma, hepatocellular carcinoma, and focal nodular hyperplasia.

Among the differential diagnoses, hepatoblastoma and undifferentiated embryonal sarcoma were considered particularly important. Hepatoblastoma usually occurs in young children and commonly appears as a predominantly solid, heterogeneous mass, sometimes containing calcification. The markedly elevated serum α-fetoprotein level in our patient initially raised concern for hepatoblastoma. Undifferentiated embryonal sarcoma generally occurs in older children and often appears as a large, predominantly cystic mass with enhancing septa or solid components [6,8]. In contrast, the lesion in the present case was predominantly multicystic, lacked calcification and diffusion restriction, and demonstrated a subtle increase in signal intensity within the solid component on hepatobiliary-phase imaging relative to the precontrast images. The patient’s age and the predominantly multicystic morphology favored mesenchymal hamartoma; however, the slight hepatobiliary-phase signal increase should be regarded as a supportive observation rather than a definitive diagnostic feature.

In the case of a hepatic mass protruding outside of the liver, lymphangiomas, duplication cysts, and anterior hepatic cysts should also be considered in the differential. Fluid retention in the mesenchyme and cyst is thought to be responsible for tumor growth, and large cysts occur as a result of mesenchymal tissue degeneration and fluid retention from the bile ducts [9]. In most mesenchymal hamartoma cases, AFP is within the normal range, and only a few cases have demonstrated increased AFP [10]. Some hepatic hamartomas that show high AFP levels histologically exhibit a morphology similar to fetal hepatocytes and contain AFP-positive hepatocytes. These cells are thought to play a role in increasing serum AFP [11]. Although high AFP levels were observed in the present case, no AFP-positive hepatocytes were identified histologically. After surgery, the AFP level decreased to the normal range, although the details of this decrease in AFP are unknown.

On CT, mesenchymal hamartoma can show various appearances depending on its internal composition. Plain CT usually depicts the tumor as a large abdominal mass with heterogeneous CT values. The interior may vary from a solid mass with small cysts that show low density to a multiloculated cystic mass with thin septa and fluid-containing spaces. Hemorrhage occurs occasionally. There have been reports of cases with calcification of the septum within the tumor [12], but this is also not typical. Dynamic studies have reported peripheral or septal enhancement, similar to hemangioendothelioma and hemangioma [13]. CT findings of our case were consistent with those of the previous reports.

On MRI, as in other modalities, the appearance differs depending on whether the solid or the cystic component is predominant. In cystic predominance, fluid accumulates in the cysts bordered by a septum that shows low T1WI/T2WI signals, and the signal intensity varies according to protein concentration. Masses with a predominantly solid component show low signal intensity on both T1- and T2-weighted images, reflecting the presence of fibrous tissue compared to the surrounding normal liver parenchyma.

Contrast enhancement with gadolinium contrast does not enhance the cystic component, but the solid component is somewhat more heterogeneous in the enhanced areas than in the normal hepatic parenchyma [14]. MR findings of our case were also consistent with those of the previous reports, except for the enhancement pattern on hepatocyte-specific contrast imaging.

Few reports have described the hepatobiliary-phase findings of gadoxetate disodium–enhanced MRI in hepatic mesenchymal hamartoma. One review noted that the enhancing portions of these tumors remain hypointense relative to the normal liver parenchyma during the hepatobiliary phase and that the cystic components show no contrast enhancement [8]. In the present case, the solid component was slightly hypointense relative to the normal liver parenchyma but became isointense to the renal cortex during the hepatobiliary phase. Because the solid component was hypointense relative to the renal cortex on the precontrast images, this change suggested a slight hepatobiliary-phase signal increase. Histopathologically, normal hepatic parenchyma was reticularly interspersed within the mass. However, immunohistochemical staining for OATP1B3 showed cytoplasmic immunoreactivity in the hepatocyte-like cells without membranous staining. Therefore, the observed signal increase may reflect delayed contrast retention within the interstitium of the solid component rather than OATP1B3-mediated hepatocellular uptake. Although speculative, a contribution from the bile duct components cannot be entirely excluded.

## Conclusion

We report a hepatic mesenchymal hamartoma showing a slight signal increase within the solid component on hepatobiliary-phase gadoxetate disodium–enhanced MRI. This finding may reflect delayed contrast retention within the interstitium rather than OATP1B3-mediated hepatocellular uptake. Although nonspecific, this finding should be recognized because a slight hepatobiliary-phase signal increase does not exclude the diagnosis of mesenchymal hamartoma. Further investigation in additional cases is needed.

## Author contributions

T.S. and N.T. contributed to manuscript writing and image interpretation. S.K. was responsible for data collection and figure preparation. M.K. and M.T. were involved in patient management, writing of the surgical findings section, and critical revision for important intellectual content. K.K. and K.A. contributed to writing of the pathology section, figure preparation, and critical revision for important intellectual content. S.H., T.O., and S.K. were involved in patient management and critical revision for important intellectual content. A.N. contributed to the conception and design of the report, overall supervision, and critical revision for important intellectual content. All authors reviewed and approved the final version of the manuscript.

## Patient consent

Written informed consent for publication of this case report and the accompanying images was obtained from the patient’s parents.
